# Bacteriological quality and prevalence of foodborne bacteria in broiler meat sold at live bird markets at Mymensingh City in Bangladesh

**DOI:** 10.5455/javar.2022.i608

**Published:** 2022-09-30

**Authors:** Shahjada Mohammad Julqarnain, Palash Bose, Md. Zaminur Rahman, Mst. Minara Khatun, Md. Ariful Islam

**Affiliations:** Department of Microbiology and Hygiene, Faculty of Veterinary Science, Bangladesh Agricultural University, Mymensingh, Bangladesh

**Keywords:** Broiler meat, bacteriological quality, live bird market, foodborne bacteria

## Abstract

**Objective:**

This study assessed the bacteriological quality and prevalence of foodborne bacteria in raw broiler meat sold in Mymensingh City.

**Materials and Methods:**

Thigh and breast meat samples (n = 80) from broiler chickens were randomly collected from four live bird markets (LBM) in Mymensingh city for bacteriological analysis. To determine the bacteriological quality, a 10-fold serial dilution of the thigh and breast homogenate was made. Then, total viable count (TVC), total coliform count (TCC), Staphylococci, and *Salmonella* spp. counts were determined using plate count agar, MacConkey agar, Mannitol salt agar, and Salmonella-Shigella agar. Gram stain, biochemical testing, PCR assays, and cultural properties were used to identify the bacterial isolates.

**Results:**

The TVC in the broiler meat sample ranged between log_10_ 8.30 ± 0.54 colony forming unit (CFU)/gm and log_10_ 9.04 ± 0.26 CFU/gm. TCC was found between log_10_ 5.53 ± 0.38 CFU/gm and log_10_ 6.66 ± 0.80 CFU/gm. The mean *Staphylococcal* count was recorded between log_10_ 4.64 ± 0.61 CFU/gm and log_10_ 6.42 ± 0.53 CFU/gm, and the total *Salmonella* count ranged between log_10_ 4.75 ± 0.08 CFU/gm and log_10_ 5.69 ± 0.58 CFU/gm. The prevalence of *Escherichia coli* was the highest (43.2%), followed by *Staphylococcus aureus* (36.8%) and *Salmonella* spp. (20%), respectively.

**Conclusions:**

Data from this study indicated that the TVC and TCC of raw broiler meat sold at LBM exceed the permissible limits and are contaminated with foodborne bacteria, which might cause public health hazards.

## Introduction

Our everyday diet includes a significant amount of broiler meat, which is much cheaper and very popular with people of all ages. Per capita per year, broiler meat consumption in Bangladesh is 3.74 kg, and its share is 54% of the total meat [1]. The adverse effects of zoonotic foodborne infections on the general public’s health are becoming increasingly well known. Several epidemiological surveys have identified foods of animal origin as the leading carriers of illnesses caused by foodborne pathogens. Getting safe food to consumers, growers, and health experts has been one of the most important and challenging things to do. Chicken meat is known to be contaminated with pathogenic and spoilage microorganisms during slaughter, skinning, and evisceration processes if adequate hygienic and sanitary measures are not undertaken [2]. Meat contamination by foodborne bacteria is a growing public health concern [3].

The timing of feed withdrawal influences the bacteriological qualities of poultry meat before slaughter, the mode of transportation, contamination from live birds, the processing method, environmental temperature, and hygienic and sanitary procedures in the slaughterhouse [4]. Depending on how the carcasses are processed at the slaughterhouses, the microbial burdens on the birds may go down or up [5]. To identify environmental and fecal contamination in chicken meat, total coliform and total fecal coliform counts can be used [6]. Total aerobic plate count can be used to determine the meat’s sanitary status, whereas total Staphylococci count and *Staphylococcus aureus* count are indicators of inadequate temperature control, handling, and hygienic conditions [7].

Several foodborne pathogens, including *Escherichia coli*, *Salmonella* spp., *S. aureus, Campylobacter* spp., Vibrio spp., and Shigella spp., can contaminate poultry flesh and infect people when handled raw or eaten undercooked [8]. The cost of medical treatment and death from foodborne illness results in economic losses [9]. In Bangladesh, around 30 million individuals contract foodborne illnesses yearly [10]. Most people in Bangladesh consumed chicken carcasses that were slaughtered and processed in small retail establishments. Without adequate temperature control, meat is sold outside, and its hygienic status is never guaranteed. Chicken meats sold at retail shops and poultry farms were found to be contaminated with an unacceptable bacterial load, which poses a public health risk to the consumers [11,12]. Production and consumption of poultry meat in Bangladesh are constantly increasing, and ensuring its microbial safety is essential. Data on the bacterial contamination of broiler meat sold at live bird marketplaces in Bangladesh is quite scarce. To ascertain the sanitary quality and hygienic state of broiler meat sold at live bird markets (LBM), the current study set out to (i) quantify the bacterial load [total viable count (TVC), total coliform count (TCC), Staphylococcus spp., and Salmonella spp.] and (ii) evaluate the prevalence of foodborne bacteria (*E. coli*, *S. aureus*, and *Salmonella* spp.).

## Materials and Methods

### Collection of broiler meat samples

**Figure 1.**
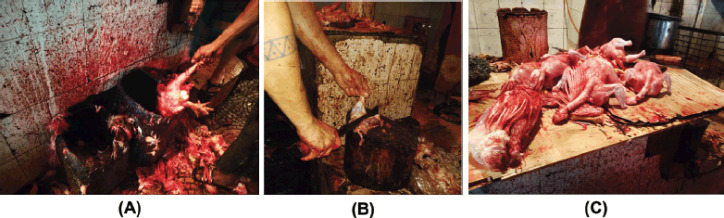
Slaughter and processing of broiler. (A) Bleeding, skinning, and evisceration of broiler carcasses at a slaughterhouse under unhygienic conditions, (B) Cutting meat using a dirty knife and wooden cutting board, and (C) Broiler carcasses were kept on the dirty floor.

Broiler meat samples from the thighs (*n* = 40) and breasts (*n* = 40) were aseptically taken from the slaughterhouses of four LBM in Mymensingh city during the winter months of November to January 2017. Twenty broiler meat samples comprised of thigh (*n* = 10) and breast (*n* = 10) were collected from each LBM. For bacteriological analysis, samples were sent to a microbiology lab in an ice box. In the live bird market, bleeding, skinning, and evisceration operations in the slaughterhouse were done under unhygienic conditions (Fig. 1A). Good hygienic practices of the poultry slaughterhouse workers were not seen. The knife and wooden cutting board used for cutting meat are not clean (Fig. 1B). Meats were frequently contaminated by the dirty floor of the slaughterhouse (Fig. 1C).

### Preparation of meat samples

Using sterile scissors, a 25 gm section of each breast and thigh sample was cut, then put into a sterile bag with 0.1% sterilized buffer peptone water [13]. Under aseptic conditions, samples were homogenized using a stomacher. The homogenate was then diluted step by step until it was diluted 10 times in 9 ml of sterile 0.1% buffer peptone water.

### Bacteriological analysis

TVC, TCC, *Staphylococcus* spp., and *Salmonella* spp. counts were estimated by dispersing one ml dilutions of each sample onto Plate count agar (HiMedia, Mumbai, India), MacConkey agar (MCA; HiMedia, Mumbai, India), Mannitol salt agar (HiMedia, Mumbai, India), and Salmonella-Shigella (SS; HiMedia, Mumbai, India) agar, respectively [6]. The inoculation plates were incubated at 37°C for 24 h. Gram staining, sugar fermentation, and biochemical tests such as catalase, oxidase, and coagulase were used to identify the *S. aureus* suspected culture, while Indole, Methyl Red (MR), Voges Proskauer (VP), catalase, Triple Sugar Iron (TSI) agar, Urea Hydrolysis, and Citrate Utilization tests were performed to confirm the presence of *E. coli* [14]. A 25 gm meat sample was enriched in 225 ml of sterile buffered peptone water and cultured at 37°C for 24 h to isolate *Salmonella* spp. An enriched sample was added to 45 ml of Selenite-F broth and incubated there for 24 h at 37°C. SS agar was streaked with a loopful of enhanced culture, which was then incubated at 37°C for 24 h [15]. The colony formed on SS agar was sub-cultured onto MCA and Nutrient agar (HiMedia, Mumbai, India) and incubated at 37°C for 24 h to get a pure *Salmonella* culture. Gram‘s staining method and the MR, VP, Indole, Oxidase, Catalase, Urea Hydrolysis, and TSI agar tests were used to confirm the presence of *Salmonella* spp.

### Molecular detection of E. coli, S. aureus, and Salmonella spp.

The previously mentioned heat lysis procedure was used to recover the DNA from *E. coli*, *S. aureus*, and *Salmonella* spp. These bacteria were molecularly confirmed using PCR assays. Table 1 shows a list of the oligonucleotide primers employed in the PCR.

### Statistical analysis

Data from the microbiological analysis were entered into a spreadsheet created with Microsoft Excel 2007. A calculation was performed to convert the mean bacterial counts [Colony forming unit (CFU)/gm] into log_10_ CFU/gm. Version 16.0 of IBM-SPSS software was used to analyze the data. One-way analysis of variance was used to compare the mean counts of the two chicken parts and the examined LBM at a 95% confidence level (*p* < 0.05).

## Results

### Mean bacterial load counts of broiler meat per live bird market

Table 2 displays the mean log_10_ CFU/gm counts of TVC, TCC, *Staphylococcus* spp., and *Salmonella* spp. in chicken meat from four live bird marketplaces. The mean counts in four live bird marketplaces did not differ significantly (*p* > 0.05).

The bacterial load was the highest at LBM-1 (log_10_ 9.04 ± 0.26 CFU/gm) and the lowest at LBM-4 (log_10_ 8.30 ± 0.54 CFU/gm). The coliform count was highest at LBM-2 (log_10_ 6.66 ± 0.80 CFU/gm) and lowest at LBM-1 (log_10_ 5.53 ± 0.38 CFU/gm). The highest counts of Staphylococci and *Salmonella* spp. were recorded from LBM-3 and LBM-4, respectively.

### Mean bacterial load counts in thigh and breast meats

Table 3 shows the mean log_10_ CFU/gm of TVC, TCC, *Staphylococcus*, and *Salmonella* spp. counts of thigh and breast meats. Thigh meat showed the highest mean bacterial count (log_10_ 5.85 ± 0.90 CFU/gm) compared to the breast (log_10_ 5.08 ± 0.43 CFU/gm). There was no significant difference in the mean log_10_ CFU/gm bacterial counts between meat from the thigh and the breast (*p* > 0.05).

**Table 1.** List of oligonucleotide primers used in the PCR assays.

Bacteria	Oligonucleotide sequence		Size (bp)	References
*E. coli*	F	5’-AATTGAAGAGTTTGATCATG-3’	704	Guan et al. [16]
	R	5’-CTCTACGCATTTCACCGCTAC-3’		
*Salmonella* spp.	F	5’-ACTGGCGTTATCCCTTTCTCTGGTG-3’	496	Cohen et al. [17]
	R	5’-ATGTTGTCCTGCCCCTGGTAAGAGA-3’		
*S. aureus*	F	5’-GCGATTGATGGTGATACGGTT-3’	279	Zhang et al. [18]
	R	5’-AGCCAAGCCTTGACGAACTAA AGC-3’		

F = Forward, R = Reverse, -bp = base pair.

**Table 2.** Mean bacterial load of chicken meat per LBM.

Sampling sites	TVC (log_10_ CFU ± SD/gm)	TCC (log_10_ CFU ± SD/gm)	*Staphylococcus* spp. (log_10_ CFU ± SD/gm)	*Salmonella* spp. (log_10_ CFU ± SD/gm)
LBM-1	8.35 ± 0.39	5.53 ± 0.38	4.64 ± 0.61	4.75 ± 0.08
LBM-2	9.04 ± 0.26	6.66 ± 0.80	6.17 ± 0.35	5.27 ± 0.53
LBM-3	8.47 ± 0.36	5.98 ± 0.42	6.42 ± 0.53	5.53 ± 0.21
LBM-4	8.30 ± 0.54	5.83 ± 0.08	6.22 ± 0.48	5.69 ± 0.58

LBM = Live Bird Market, TVC: Total Viable Count, TCC: Total Coliform Count, CFU = Colony Forming Unit, SD = Standard Deviation.

### Isolation and molecular detection of bacteria from broiler meat

**Figure 2.**
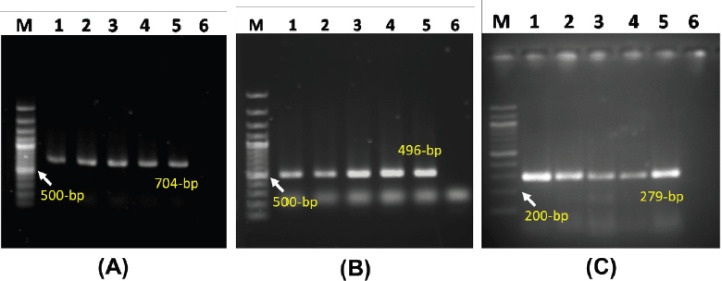
PCR-detection of *E. coli*, *Salmonella*, and *Staphylococcus* spp. (A) Amplification of 704-bp fragment of *16SrRNA* gene of *E. coli*., (B) Amplification of 496-bp fragment of *16S rRNA* gene of *Salmonella* spp., and (C) Amplification of 279-bp fragment of *nuc* gene of *S. aureus*. In all cases, Lane 1-4: DNA samples, Lane M: 100-bp size DNA marker, Lane 5: positive control, and Lane 6: negative control.

*E. coli* (*n =* 54), *S. aureus* (*n =* 46), and *Salmonella* spp. (*n =* 25) were isolated and identified using routine bacteriological methods and PCR assays (Fig. 2).

### Prevalence of foodborne bacteria in broiler meat

The *E. coli* isolates recorded the highest (43.2%), followed by *S. aureus* (36.8%) and *Salmonella* spp. (20%). The highest level of bacterial prevalence was noted in broiler meat at LBM-3 (32%), followed by LBM-4 (24.8%), LBM-2 (23.2%), and LBM-1 (20%) (Table 4).

Table 5. shows the highest prevalence of *E. coli* both in the thigh (77.5%) and breast (57.5%) meat, followed by *S. aureus* (67.5% and 47.5%) and *Salmonella* spp. (45% and 17.5%). There was no big difference in the number of bacterial isolates between the thigh and breast meat (*p* > 0.05).

## Discussion

In Bangladesh, most poultry is slaughtered in temporary slaughterhouses at the LBM adjacent to the road and crowded market places, which often lack water supply for washing hands and cleaning, cutting knives, and utensils. Furthermore, workers in temporary slaughterhouses lack adequate knowledge of personal hygiene and sanitation procedures, resulting in bacterial contamination of poultry carcasses. The quality of meat is assessed by counting TVC, and it also indicates the food safety status of meat. This study recorded TVC of > log_10_ 8 CFU/gm. Murshed et al. [19] also noted a comparable mean TVC log count (log_10_ 8.46 CFU/gm) in raw chicken meat. As compared to this study, a lower TVC for market chicken meat was reported in Nepal (log_10_ 4.45 CFU/gm) [6], Pakistan (log_10_ 5.07CFU/gm) [20], and Egypt (log_10_ 6.18 CFU/gm) [21]. TVC indicates the hygienic condition of the slaughterhouse where chickens are slaughtered and processed. The meat‘s higher TVC count (107 CFU/gm) is responsible for microbial spoilage and off-odor [22]. The log TVC count in this study exceeds the permissible level (log_10_ ≥6 CFU/gm) [23], indicating very poor hygienic practices at the slaughterhouses at the LBM.

**Table 3.** Mean bacterial counts of thigh and breast meats.

Chicken parts	TVC (log_10_ CFU ± SD/gm)	TCC (log_10_ CFU ± SD/gm)	*Staphylococcus* spp. (log_10_ CFU ± SD/gm)	*Salmonella* spp. (log_10_ CFU ± SD/gm)
Thigh	8.25 ± 0.81	6.78 ± 0.87	6.15 ± 0.92	5.85 ± 0.90
Breast	8.11 ± 0.78	5.77 ± 0.44	6.05 ± 0.88	5.08 ± 0.43

TVC = Total Viable Count, TCC = Total Coliform Count, CFU = Colony Forming Unit, SD = Standard Deviation.

**Table 4.** Prevalence of foodborne bacteria per LBM.

Bacterial isolates	Sampling sites				Total (*N/%)
	LBM-1 (*n/%)	LBM-2 (*n/%)	LBM-3 (*n/%)	LBM-4 (*n/%)	
*E. coli*	13 (65.0)	11(55)	18 (90)	12(60.0)	54(43.2)
*S. aureus*	8 (25.0)	12 (60.0)	15(75.0)	11(55.0)	46(36.8)
*Salmonella* spp.	4 (20.0)	6 (30.0)	7 (35.0)	8(40.0)	25(20.0)
Total (%)	25 (20.0)	29(23.2)	40 (32.0)	31(24.8)	125 (100)

LBM = Live Bird Market, *n = 20, *N = 125.

**Table 5.** Prevalence of foodborne bacteria in thigh and breast meats.

Bacterial isolates	Thigh (*n/%)	Breast (*n/%)	Total (*N/%)
*E. coli*	31 (77.5)	23 (57.5)	54 (43.2)
*S. aureus*	27 (67.5)	19 (47.5)	46 (36.8)
*Salmonella* spp.	18 (45.0)	7 (17.5)	25 (20.0)
Total (%)	76 (60.8)	49(39.2)	125 (100)

*n = 40, *N = 125.

This study recorded TCC between log_10_ 5.53 ± 0.38 CFU/gm and log_10_ 6.66 ± 0.80 CFU/gm, similar to the TCC (log_10_ 6.5 CFU/gm) reported by Bhandari et al. [24]. As compared to the present study, a lower TCC in chicken meat was reported in Bangladesh (log_10_ 3.37 CFU/gm), India (log_10_ 1.13 CFU/gm), and Nepal (log_10_ 2.19 CFU/gm) by Murshed et al. [19], Selvan et al. [25], and Maharjan et al. [6], respectively. This study recorded higher TCC in the thigh meat (log_10_ 6.78 ± 0.87 CFU/gm) as compared to breast meat (log_10_ 5.77 ± 0.44 CFU/gm) since the thigh area is more exposed to fecal contamination during handling and processing of the carcass [19]. In the present study, the TCC in the raw broiler meat sold at the LBM exceeds the acceptable limit (≤100 CFU/gm) prescribed by the International Commission on Microbiological Specifications of Foods [23]. The presence of higher coliform counts in the broiler meat sold at the live bird market might result from fecal contamination during slaughter operations, evisceration, as well as poor personal hygiene of the slaughterhouse workers.

In this study, foodborne bacteria like *E. coli*, *Salmonella* spp., and *S. aureus* isolated from broiler meat were confirmed by PCR assays. *E. coli., Salmonella spp.,* and *S. aureus* recovered from thigh and breast meat samples successfully amplified 704-bp, 496-bp and 274-bp PCR amplicons, which confirmed their identity at the molecular level [16–18].

Meat containing the *E. coli* indicator organism is likely to be contaminated with feces and indicates poor hygiene [26]. In this investigation, the prevalence of *E. coli* varied between 43.2% and 90%. Elzaher et al. [27] showed a similar incidence of *E. coli* (87.5%) in poultry flesh. In this study, thigh meat had a greater prevalence of *E. coli* (77.5%) than breast meat (57.5%). Both thigh meat (9%) and breast meat (12%) in Egypt were found to have lower *E. coli* prevalence [28]. A study conducted in Romania recorded a 34% prevalence of E. coli in poultry carcasses [29].

This study recorded a total *Salmonella* count of > log_10_ 4.75 CFU/gm in raw broiler meat, while Uddin et al. [30] found *Salmonella* spp. counts between log_10_ 1.3 CFU/gm and log_10_ 2.67 CFU/gm in chicken meat sold at super shops in Dhaka, Bangladesh. *Salmonella* was detected in poultry flesh between 20% and 40% in this study. The *Salmonella* prevalence in Nepali retail broiler meat ranged from 10% to 46.2% [24,31]. Research shows a 19%–24% prevalence of *Salmonella* contamination in retail [32]. According to Cretu et al. [29], there was a 2% prevalence of *Salmonella* on the surface of chicken carcasses. In Pennsylvania, 19% of the prepared broiler meat sold in grocery stores had *Salmonella* contamination [32]. In the present study, the prevalence of *Salmonella* was higher in thigh meat (45%) than in breast meat (17.5%). Guran et al. [33] reported a higher prevalence of *Salmonella* both in breast meat (44.7%) and thigh meat (41%). *Salmonella* could get into the meat from feces or the hands of the butcher when he washed and cut it [34,35].

In the present study, the Staphylococci count of raw broiler meat ranged between log_10_ 4.64 ± 0.61 CFU/gm and log_10_ 6.42 ± 0.53 CFU/gm, which was higher than the Staphylococci count recorded by Sengupta et al. [36] (log_10_ 3.7 CFU/gm) and Joshi et al. [37] (log_10_ 4.07 CFU/gm). *Staphylococcus aureus* contaminates the meat from unhygienic slaughterhouses while processing and handling the carcass. According to Carroll et al. [38], *S. aureus*, which causes foodborne poisoning, is a natural flora of the human skin, respiratory system, external ear, and mouth. Their presence in the food samples was mainly due to the unwholesome practices of the handlers. In this investigation, *S. aureus* prevalence ranged from 25% to 75%. In Romania, 14% of poultry carcasses were contaminated with *S. aureus* [29]. Herve et al. [39] reported 53% of *S. aureus* contamination in retail chicken meat. *Staphylococcus aureus* prevalence in retail meat in China was reported by Wu et al. [40] to be 35%. Contamination of chicken meat at the slaughterhouse resulted from the sneezing, coughing, breathing, and talking of infected people [6]. In this study, TCC and total Staphylococci and *Salmonella* counts were higher than TVC. Similar results were reported in cattle sold at retail markets [41]. A study conducted in Nepal recorded almost similar TCC (8.13 ± 0.13) and TVC (8.22 ± 0.14) in raw meat [42].

## Conclusion

The study‘s data indicate that the bacterial load in raw broiler meat at the LBM exceeds the permissible limit. To minimize the present bacterial burden to an acceptable level, appropriate sanitary and hygienic practices at the broiler slaughterhouses need to be adopted. A higher prevalence of foodborne bacteria in the raw broiler meat at the live bird markets might cause a health risk to slaughterhouse staff and meat consumers. Undertaking appropriate intervention measures, including training programs for slaughterhouse workers and building consumer awareness, is required to mitigate this health risk.

## List of abbreviations

LMB, live bird market; PCR, polymerase chain reaction; TVC, total viable count, TCC, total coliform count, MCA, MacConkey agar; CFU, colony forming unit.
